# Serum lactate and carboxyhemoglobin as predictors of hyperbaric oxygen therapy in carbon monoxide poisoning: a retrospective study

**DOI:** 10.1186/s12873-025-01410-w

**Published:** 2025-12-03

**Authors:** Ahmet Aykut, Ertuğ Günsoy, Burcu Özen Karabulut, Ramazan Sami Aktaş, Mehmet Veysel Öncül, Ali Ekber Karabulut

**Affiliations:** 1 Department of Emergency Medicine, SBU. Van Education and Research Hospital, Van, Turkey; 2 Department of Emergency Medicine, Ağrı İbrahim Çeçen University, Ağrı, Turkey

**Keywords:** Carbon monoxide poisoning, Lactate, Carboxyhemoglobin, Hyperbaric oxygen therapy, Triage, Hematological parameters

## Abstract

**Background:**

Carbon monoxide (CO) poisoning is a leading cause of toxicological emergencies worldwide. Although carboxyhemoglobin (COHb) levels are traditionally used to confirm exposure, they often fail to reflect clinical severity. This study aimed to evaluate the diagnostic performance of serum lactate and hematological parameters in predicting the need for hyperbaric oxygen therapy (HBOT) among patients with acute CO poisoning.

**Materials and methods:**

This retrospective cross-sectional study included 292 adult patients with confirmed CO poisoning admitted to a tertiary emergency department between 2020 and 2024. Patients were categorized according to HBOT administration. Laboratory values including lactate, COHb, and hematologic parameters and indices were analyzed. Multivariable logistic regression and ROC curve analysis were used to assess predictive performance.

**Results:**

Of the 292 patients, 94 (32.2%) received HBOT. Serum lactate and COHb were significantly higher in the HBOT group (*p* < 0.001 for both) and were identified as independent predictors of HBOT requirement (lactate OR = 2.04; COHb OR = 1.32). AUC for lactate alone was 0.754; combining lactate with hematologic markers modestly improved AUC to 0.769. The most robust model, incorporating lactate and COHb, achieved an AUC of 0.936. Hematologic markers alone showed limited predictive value.

**Conclusion:**

Serum lactate, particularly when combined with COHb, provides strong diagnostic value in predicting HBOT need in CO poisoning. The integration of these readily available biomarkers may improve triage decisions in emergency care.

## Background

Carbon monoxide (CO) poisoning remains a major cause of toxicological emergencies worldwide, with potentially fatal consequences due to its ability to impair oxygen delivery and induce systemic hypoxia. CO has over 200-fold greater affinity for hemoglobin than oxygen, leading to the formation of carboxyhemoglobin (COHb), which compromises oxygen transport and tissue oxygenation [1]. In addition, CO binds to mitochondrial cytochrome c oxidase, inhibiting the electron transport chain and disrupting oxidative phosphorylation. This mitochondrial dysfunction triggers a shift to anaerobic glycolysis, resulting in intracellular acidosis and elevated lactate production—linking lactate directly to the pathophysiology of CO toxicity [2].

Traditionally, COHb levels have been used to confirm exposure and estimate severity. However, several studies have shown that COHb concentrations are not consistently predictive of clinical outcomes, especially in cases with delayed presentation or comorbid conditions [2]. Moreover, COHb levels rapidly decline with oxygen therapy, limiting their utility in guiding acute treatment decisions [3]. As such, alternative biomarkers with greater pathophysiological relevance and real-time availability are needed to inform management decisions such as hyperbaric oxygen therapy (HBOT) initiation.

Lactate has emerged as a promising candidate in this context. It reflects anaerobic metabolism and tissue hypoxia, both of which are central to CO-induced injury (4). Elevated serum lactate levels have been associated with more severe clinical presentations and worse outcomes in CO poisoning [3]. Additionally, routine hematologic parameters—including white blood cell count (WBC), neutrophil-to-lymphocyte ratio (NLR), platelet-to-lymphocyte ratio (PLR), mean platelet volume (MPV), and red cell distribution width–coefficient of variation (RDW-CV)—have been proposed as surrogate markers of systemic inflammation and oxidative stress in toxicological emergencies [4, 5].

This study aimed to evaluate the diagnostic value of serum lactate and selected hematologic indices in predicting the need for HBOT among patients presenting with acute CO poisoning. By identifying simple and accessible biomarkers, we hope to improve early risk stratification and guide timely therapeutic interventions in the emergency setting [6].

## Materials and methods

### Study design and settings

This retrospective, cross-sectional study was conducted at the Emergency Department of Van Education and Research Hospital (Health Sciences University–SBU), a tertiary care, university-affiliated center in Van, Türkiye. The aim was to evaluate the predictive value of serum lactate and selected hematological parameters in determining the need for HBOT in patients presenting with acute CO poisoning. The study included patients admitted between January 1, 2020, and December 31, 2024.

### Ethical approval and data privacy

Ethical approval was obtained from the institutional ethics committee (Approval No: GOKAEK/2025-03-07; Date: March 28, 2025). All procedures were conducted in accordance with the Declaration of Helsinki. Patient data were anonymized prior to extraction, and no identifiable information was used in the analysis. As the study was retrospective and non-interventional, the requirement for informed consent was waived. This study complies with the STROBE (Strengthening the Reporting of Observational Studies in Epidemiology) guidelines for observational research.

### Patient selection

Patients were eligible for inclusion if they were 18 years of age or older, had a diagnosis of CO poisoning confirmed by COHb level > 5%, and had venous blood gas analysis and complete blood count results available at initial presentation. Patients were excluded if they were pregnant, transferred from another facility, had incomplete laboratory or clinical documentation, or were suspected of co-exposure to other toxic agents, such as cyanide, particularly in the context of fire-related inhalation injury. While prehospital treatments such as supplemental oxygen administration by emergency medical services were frequently implemented, they were not consistently documented in the medical records and could not be accounted for in the analysis. This potential source of confounding is acknowledged in the study limitations.

### Outcome definition

The primary outcome was defined as the administration of HBOT during the index encounter. At the study institution, HBOT is indicated based on an internal clinical protocol and multidisciplinary assessment. Criteria include a COHb level greater than 25%, the presence of neurological symptoms such as confusion, syncope, or seizures, evidence of myocardial ischemia on electrocardiogram or elevated cardiac biomarkers, persistent symptoms despite normobaric oxygen therapy, and risk-enhancing features such as advanced age or comorbid disease. Final treatment decisions were made following consultation with board-certified specialists in Undersea and Hyperbaric Medicine, in accordance with institutional protocol. Emergency physicians initiated the referral process, and the indication for HBOT was evaluated by the hyperbaric medicine team based on clinical presentation and laboratory findings. While HBOT administration was used as a surrogate for clinical severity in this study, we acknowledge that it may not fully reflect uniform disease burden due to institutional or provider-level variability. Accordingly, the outcome represents a clinician decision under real-world practice rather than a patient-centered clinical endpoint, and our models aim to predict this decision-making behavior.

### Data collection

Demographic data (age and sex), initial laboratory parameters, and exposure-related biomarkers were extracted from electronic medical records. Laboratory values obtained within the first 30 min of emergency department admission included venous lactate (mmol/L), COHb (%), WBC, neutrophil count (NEU), lymphocyte count (LYM), platelet count (PLT), RDW-CV, and MPV. Two derived ratios were also calculated: NLR and PLR. Markers of end-organ injury such as cardiac troponin and neurological status assessments (e.g., Glasgow Coma Scale) were not consistently recorded across cases and were therefore excluded from analysis. This limitation is discussed further in the Discussion section.

Because numeric GCS and troponin values were inconsistently recorded as structured fields, we excluded them from the primary regression analyses. For clinical severity grading, however, we abstracted the worst documented clinical state (e.g., syncope, confusion, seizure, coma, respiratory failure/intubation, vasopressor use, arrhythmia or MI) from narrative notes and disposition records and assigned the Poison Severity Score (PSS) (0 none, 1 minor, 2 moderate, 3 severe, 4 fatal) accordingly. When essential elements were unavailable, PSS was coded as missing and excluded from PSS-specific analyses.

### Sample size consideration

Sample size calculation was based on prior literature suggesting that lactate has approximately 77% sensitivity in predicting the need for HBOT in CO poisoning. Assuming a prevalence of HBOT use in 30% of cases, a minimum of 227 patients was required to estimate sensitivity with a 10% margin of error at a 95% confidence level. A separate estimate determined that 97 patients were necessary to evaluate specificity. The final study sample (*n* = 292) met both criteria.

### Statistical analysis

We pre-specified COHb and lactate as primary predictors and CBC-derived indices (WBC, NEU, LYM, PLT, RDW-CV, MPV) with NLR as exploratory candidates, to test incremental value beyond COHb and lactate rather than to propose hematologic markers as stand-alone triggers.

Distributional assumptions for continuous variables were assessed using Shapiro–Wilk tests and Q–Q plots. Normally distributed variables with approximately equal variances (Levene’s test) were compared using the independent-samples t-test; when variance homogeneity was violated, the Welch t-test was applied. Markedly skewed variables (e.g., lactate, NLR, PLR) were summarized as median [IQR] and compared using the Mann–Whitney U test. Categorical variables were compared using the χ² test (or Fisher’s exact test when expected counts < 5). All tests were two-sided with α = 0.05. A multivariable logistic regression model was fitted to identify independent predictors of HBOT administration, including lactate, COHb, WBC, NEU, LYM, RDW-CV, MPV, PLT, and NLR; results are reported as odds ratios (ORs) with 95% confidence intervals (CIs). Model discrimination was quantified by the area under the ROC curve (AUC) with bootstrap 95% CIs (2,000 resamples). To evaluate incremental value, we fitted nested models (COHb only; lactate only; COHb + lactate) and compared them using ΔAUC, the likelihood-ratio test (LRT) for nested models, and ΔAIC/ΔBIC; ORs were expressed per 1% COHb and per 1 mmol/L lactate. For clinical interpretability of lactate, we reported the Youden-index cut-point and pre-specified fixed thresholds (≥ 2.5, ≥ 3.0, ≥ 4.0 mmol/L) with sensitivity and specificity. Analyses used complete-case data without imputation; PSS-based summaries were descriptive and restricted to patients with available PSS. Statistical analyses were performed in IBM SPSS Statistics v26, with bootstrap/ROC procedures confirmed in Python (scikit-learn).

## Results

### Patient characteristics

**Fig. 1:**
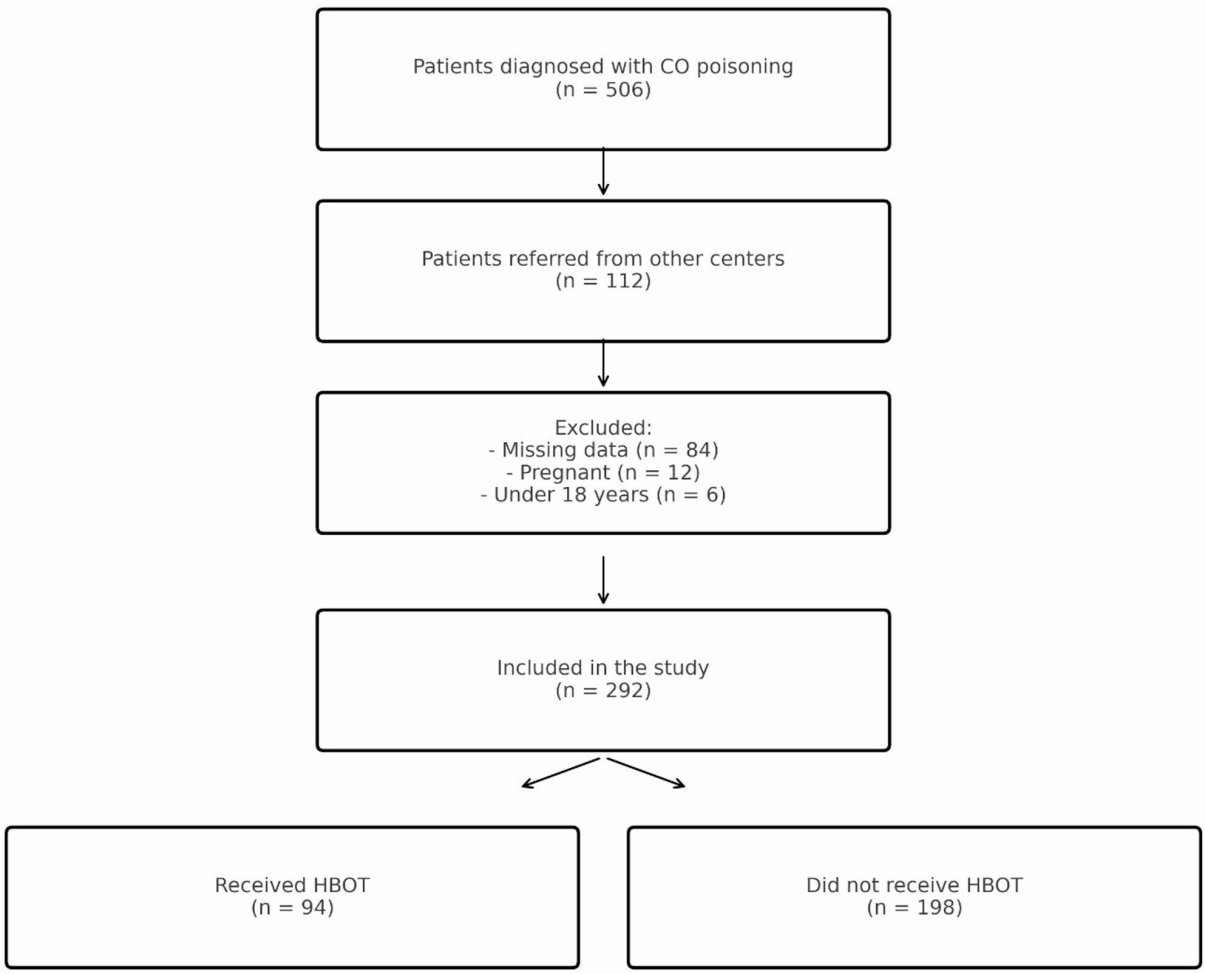
Flow diagram showing patient selection, exclusion criteria, and final grouping based on hyperbaric oxygen therapy (HBOT) status

A total of 506 patients diagnosed with CO poisoning between January 1, 2020, and December 31, 2024, were screened. After applying eligibility criteria, 292 patients were included in the final analysis (Fig. 1). Of these, 94 (32.2%) received HBOT, while 198 (67.8%) were treated with normobaric oxygen only.

The mean age of the overall cohort was 38.1 ± 15.7 years, with no significant difference between the HBOT and non-HBOT groups (*p* = 0.120). Among 170 female patients, 45 (26.5%) received HBOT, while 49 of 122 males (40.2%) underwent HBOT, showing a significant sex-based difference (*p* = 0.019). Mean COHb levels were significantly higher in the HBOT group (31.2 ± 8.0%) than in the non-HBOT group (16.1 ± 7.2%, *p* < 0.001) (Table 1).

**Table 1:** Demographics and laboratory indices by HBOT status

Characteristic	Overall (*n* = 292)	Non HBOT (*n* = 198)	HBOT (*n* = 94)	*p*-value
Age, years (mean ± SD)	38.1 ± 15.7	37.0 ± 14.6	40.3 ± 17.6	0.120
Sex, female — n (%)	170 (58.2)	125	45	0.019
Sex, male — n (%)	122 (41.8)	73	49	
**Hematological Parameters (mean ± SD or median [IQR])**				
WBC (×10^9/L)	8.76 [7.54–11.16]	8.46 [7.47–10.66]	9.96 [7.73–12.40]	0.004
NEU (×10^9/L)	6.00 [4.73–7.98]	5.96 [4.74–7.38]	6.12 [4.72–9.07]	0.142
LYM (×10^9/L)	2.09 [1.50–2.82]	2.07 [1.47–2.76]	2.17 [1.53–3.04]	0.205
RDW-CV (%)	13.30 [12.80–13.90]	13.30 [12.80–13.90]	13.30 [12.80–14.00]	0.892
MPV (fL)	10.0 ± 1.1	10.1 ± 1.2	9.9 ± 1.0	0.248
PLT (×10^9/L)	254.50 [211.00–301.50]	254.50 [211.25–296.25]	254.00 [211.25–308.75]	0.951
**Core biomarkers (mean ± SD or median [IQR])**				
Lactate (mmol/L)	2.05 [1.58–2.80]	1.90 [1.42–2.40]	2.90 [2.02–4.20]	0.000
COHb (%)	20.9 ± 10.3	16.1 ± 7.2	31.2 ± 8.0	0.000
**Hematological Indices (median [IQR])**				
NLR	2.96 [1.84–4.52]	2.91 [2.03–4.21]	3.10 [1.46–5.49]	0.838
PLR	121.57 [88.77–161.92]	123.81 [93.67–164.98]	113.39 [84.73–155.57]	0.081

Continuous variables are summarized as mean ± SD (Welch’s t-test) or median [IQR] (Mann–Whitney U) as appropriate; categorical variables by χ² test; all tests two-sided with *p* < 0.05 considered significant

HBOT, hyperbaric oxygen therapy; COHb, carboxyhemoglobin; WBC, white blood cell; NEU, neutrophil; LYM, lymphocyte; RDW-CV, red cell distribution width–coefficient of variation; MPV, mean platelet volume; PLT, platelet count; NLR, neutrophil-to-lymphocyte ratio; PLR, platelet-to-lymphocyte ratio

### Clinical severity & exposure characteristics

PSS was calculable in 214/292 (73.3%) patients. The distribution was PSS0 14 (6.5%), PSS1 138 (64.5%), PSS2 53 (24.8%), PSS3 9 (4.2%), and PSS4 0. The proportion receiving HBOT increased with severity: 7.1% for PSS0, 26.8% for PSS1, 75.5% for PSS2, and 77.8% for PSS3 (χ² *p* < 0.001).

Among records with available data (*n* = 214), exposure was accidental in 212 (99.1%) and intentional/self-harm in 2 (0.9%). The most frequent sources were coal stoves 135/214 (63.1%) and natural gas appliances 44/214 (20.6%), followed by tandoor/oven 17/214 (7.9%), fire smoke inhalation 11/214 (5.1%), and hookah/charcoal shisha 7/214 (3.3%).

### Comparison of lactate and inflammatory indices

Lactate levels were significantly elevated in patients receiving HBOT (median 2.90 mmol/L [IQR: 2.02–4.20]) compared to those who did not (median 1.90 mmol/L [IQR: 1.42–2.40]; *p* < 0.001). No significant differences were found for NLR (*p* = 0.838) or PLR (*p* = 0.081) between groups (Table 1).

### Hematological parameters

Among the hematological markers, WBC was higher in the HBOT group (*p* = 0.004), whereas NEU (*p* = 0.142) and LYM (*p* = 0.205) did not differ significantly. No significant differences were observed for RDW-CV (*p* = 0.892), MPV (*p* = 0.248), or PLT (*p* = 0.951) (Table 1).

### Predictors of HBOT requirement

A multivariable logistic regression model incorporating lactate, COHb, and hematological parameters identified both lactate (OR = 2.04; 95% CI: 1.28–3.24; *p* = 0.003) and COHb (OR = 1.32; 95% CI: 1.23–1.43; *p* < 0.001) as independent predictors of HBOT administration. None of the hematological variables were significant in the adjusted model.

### Diagnostic performance of lactate

**Fig. 2:**
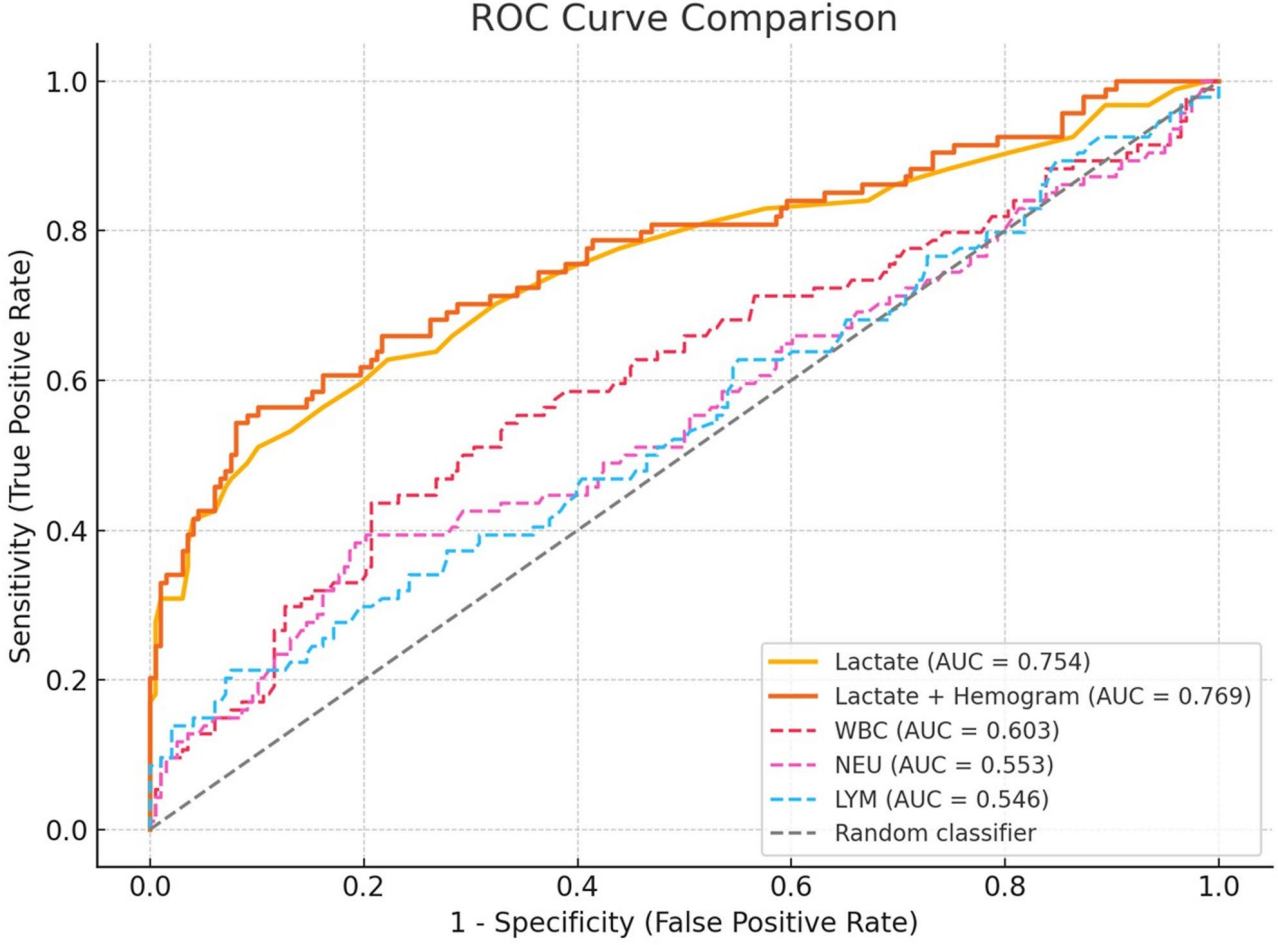
Receiver operating characteristic (ROC) curves for lactate alone, hematological parameters, and their combination in predicting hyperbaric oxygen therapy (HBOT) requirement

The ROC analysis for lactate alone yielded an AUC of 0.754 (95% CI: 0.689–0.817), indicating moderate discriminative power (Fig. 2). While the Youden index identified a predicted probability cut-off of 0.507 (not a raw lactate value), this threshold lacks direct clinical interpretability.

To support practical decision-making, fixed lactate thresholds were also evaluated. As shown in Table 2, a threshold of ≥ 2.5 mmol/L achieved a balanced sensitivity (62.8%) and specificity (77.8%), whereas ≥ 3.0 mmol/L improved specificity (90.9%) at the cost of lower sensitivity (48.9%). A high threshold of ≥ 4.0 mmol/L yielded near-perfect specificity (99.0%) but would miss more than two-thirds of patients who ultimately received HBOT.

**Table 2:** Diagnostic performance of fixed lactate thresholds for predicting HBOT requirement

Lactate Threshold (mmol/L)	Sensitivity (%)	Specificity (%)
1.5	88.3	25.3
2.0	77.7	56.1
2.5	62.8	77.8
3.0	48.9	90.9
4.0	30.9	99.0

Sensitivity and specificity values are shown for selected lactate cut-off points. A threshold of ≥ 2.5 mmol/L provided balanced diagnostic performance, while ≥ 4.0 mmol/L demonstrated high specificity but low sensitivity

### Combined model performance

**Fig. 3:**
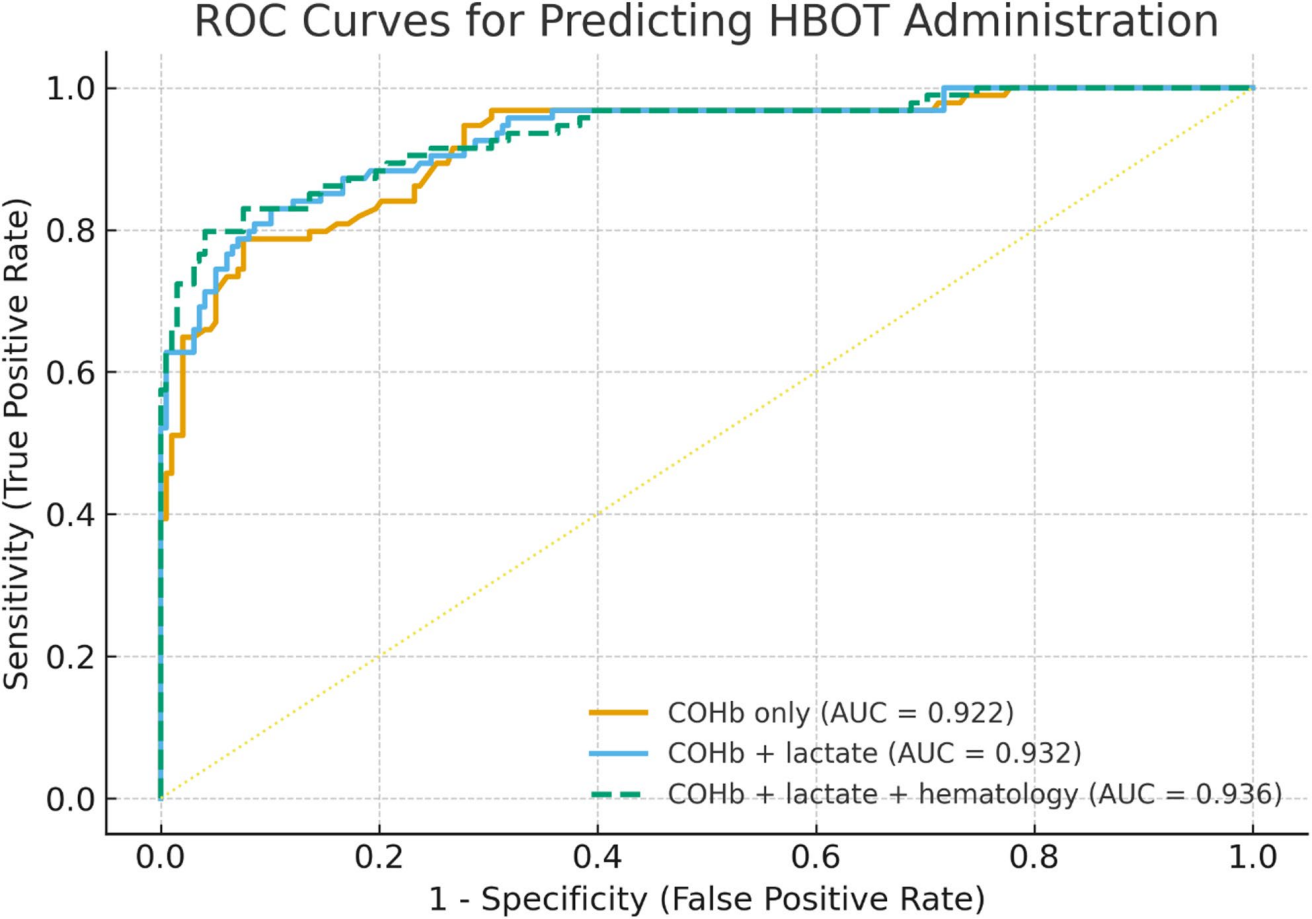
ROC curves for three nested models predicting HBOT administration: COHb alone (AUC 0.922), COHb + lactate (AUC 0.932), and COHb + lactate + hematologic indices (AUC 0.936). Adding lactate to COHb produced a small but statistically significant gain (ΔAUC 0.011; LRT χ²=16.77, *p* = 4.22 × 10⁻⁵); hematologic indices offered minimal additional discrimination

The AUC for COHb alone was 0.922 (95% CI 0.884–0.954); for lactate alone, 0.754 (0.686–0.816); and for the combined model (COHb + lactate), 0.932 (0.898–0.962) (Fig. 3). Adding lactate to COHb provided a modest but statistically significant improvement (ΔAUC = 0.011; LRT χ² = 16.77, *p* = 4.22 × 10⁻⁵), with better information criteria (AIC 193.1 → 178.3; BIC 200.4 → 189.3) (Table 3). In the combined model, COHb was associated with HBOT (OR per 1% = 1.298, 95% CI 1.211–1.390; *p* < 0.001) and lactate remained independently associated (OR per 1 mmol/L = 2.175, 95% CI 1.399–3.383; *p* < 0.001).

**Table 3:** Lactate, NLR, and PLR values by HBOT status

Model	AUC	95% CI	ΔAUC vs. COHb-only	AIC	BIC
Lactate Only	0.754	0.685–0.815	−0.168	298.3	305.7
COHb Only	0.922	0.884–0.953	0	193.1	200.5
COHb + Lactate	0.932	0.898–0.961	0.01	178.3	189.4
Lactate + Hematologic Indices	0.771	0.708–0.831	−0.151	302.3	335.4
COHb + Lactate + Hematologic Indices	0.936	0.904–0.965	0.015	182.6	219.3

Values are median [IQR] unless otherwise indicated. p-values are from Mann–Whitney U tests for continuous variables and χ² tests for categorical variables; two-sided, *p* < 0.05 considered significant

HBOT, hyperbaric oxygen therapy; NLR, neutrophil-to-lymphocyte ratio; PLR, platelet-to-lymphocyte ratio.

When lactate was combined only with hematological indices (WBC, NEU, LYM, RDW-CV, MPV, PLT, NLR), discrimination improved modestly (AUC ≈ 0.769). Adding these indices on top of COHb + lactate yielded AUC 0.936 (95% CI 0.904–0.965), a minimal additional change compared with COHb + lactate (Fig. 3). Given their limited incremental value and lack of independent significance in adjusted models, we report hematological indices within Table 3 and keep the narrative focused on COHb and lactate, which drive performance.

## Discussion

Carbon monoxide poisoning triggers a complex cascade of physiological disturbances, most notably tissue hypoxia and systemic inflammation, which are reflected in various laboratory biomarkers [2, 7]. Among these, serum lactate is widely accepted as a sensitive and early marker of anaerobic metabolism [8]. Our results confirm that lactate levels were significantly higher in patients requiring HBOT, and multivariable analysis identified lactate as an independent predictor of HBOT administration (OR = 2.04; 95% CI: 1.28–3.24) [9]. These findings are consistent with prior work demonstrating lactate’s prognostic utility in CO poisoning and its association with poisoning severity and outcome [6, 9].

Traditionally, COHb levels have guided clinical decisions in CO poisoning. However, COHb has several limitations: its levels rapidly decline with oxygen therapy, and they do not always correlate well with the extent of tissue hypoxia or the likelihood of delayed neurological sequelae [6, 9]. In contrast, lactate is a more direct biochemical indicator of cellular hypoxia and mitochondrial dysfunction [2, 4]. While COHb is still useful as an exposure biomarker, its diagnostic value improves substantially when interpreted in combination with lactate. In our study, COHb also emerged as an independent predictor of HBOT need (OR = 1.32; 95% CI: 1.23–1.43), suggesting that a multimodal approach yields superior accuracy.

This is further supported by our ROC analysis. Lactate alone demonstrated moderate discriminatory performance (AUC = 0.754; 95% CI: 0.689–0.817). When hematological parameters were added to the model, the AUC increased modestly to 0.769. However, the most significant improvement occurred when both lactate and COHb were included: the full model achieved an AUC of 0.936 (95% CI: 0.904–0.965), indicating excellent diagnostic accuracy and potential clinical applicability in triage decisions [3]. These results should be interpreted in light of the fact that HBOT is a discretionary intervention; thus, the models primarily characterize decision patterns influenced by clinical data rather than biological risk alone.

We included complete blood count (CBC)-derived indices because they are universally available at emergency department arrival and pathophysiologically plausible (systemic inflammation, erythrocyte morphology) in CO poisoning. However, in our data they did not retain independent predictive value and offered minimal incremental discrimination beyond COHb and lactate (AUC ≈ 0.771 for lactate + hematology; 0.936 vs. 0.932 when added on top of COHb + lactate, with worse information criteria). Possible explanations include outcome definition (HBOT as a decision-level endpoint), timing variability (sampling after oxygen therapy), limited dynamic range, and collinearity with primary markers. Collectively, these findings suggest CBC indices may provide contextual rather than decisional information for HBOT; we therefore deemphasize them in conclusions and report them for transparency.

To enhance real-world usability, we also assessed fixed lactate thresholds. A cut-off of ≥ 2.5 mmol/L achieved a balanced sensitivity (62.8%) and specificity (77.8%), while ≥ 3.0 mmol/L improved specificity to 90.9% with a trade-off in sensitivity (48.9%). In contrast, a very high threshold such as ≥ 4.0 mmol/L offered excellent specificity (99.0%) but would fail to detect a large proportion of at-risk patients. These findings suggest that lactate values in the 2.5–3.0 mmol/L range may serve as practical decision points to trigger enhanced monitoring or consideration for HBOT [6].

From a mechanistic standpoint, lactate production results from CO-mediated inhibition of cytochrome c oxidase in the mitochondrial electron transport chain, which disrupts oxidative phosphorylation and drives anaerobic glycolysis [2, 7]. This mechanistic linkage enhances lactate’s credibility as a biomarker rooted in CO’s pathophysiology, rather than a nonspecific stress marker. Accordingly, early measurement of lactate at the point of emergency department arrival may provide a valuable opportunity for timely intervention [8].

Although hematologic indices such as WBC, NEU, and LYM differed significantly between groups, they did not retain independent predictive power in multivariable models. RDW-CV, MPV, PLT, NLR, and PLR also lacked discriminative performance, as evidenced by low AUC values. These results are consistent with prior findings suggesting that hematologic parameters may reflect systemic inflammation or stress, but do not directly correspond with CO poisoning severity [3–5, 10–12]. Nevertheless, these routinely available markers may contribute supportive contextual information, particularly in settings where lactate or COHb testing is delayed [5].

One additional finding was the sex-based disparity in HBOT administration, with male patients more likely to receive treatment (40.2% vs. 26.5%, *p* = 0.014). While this may reflect differences in exposure intensity, occupational risks, or physician perception, further investigation is needed to understand the clinical implications of this trend.

This retrospective, single-center study cannot establish causality and is susceptible to selection and information bias. Importantly, the primary outcome—HBOT administration—represents a clinician decision and is partially informed by the very predictors evaluated (e.g., COHb, and in some cases lactate). This incorporation/decision-dependence bias may inflate apparent discrimination and should temper interpretation of the AUC. Prehospital care (notably oxygen therapy) and the timing of sampling relative to exposure and treatment were not uniformly documented, introducing potential measurement variability in initial COHb or lactate values. PSS could be calculated in 214/292 (73.3%) patients and was abstracted from narrative notes; therefore, missingness and possible misclassification are acknowledged. Although adding lactate to COHb yielded a statistically significant improvement in discrimination, the absolute ΔAUC was small (0.011) and its clinical impact may be modest. Hematologic indices were included because they are universally available and pathophysiologically plausible, yet they did not retain independent predictive value and provided minimal incremental information beyond COHb and lactate; these analyses should be considered exploratory. Finally, we did not assess long-term outcomes (e.g., delayed neurologic sequelae), and no external validation was performed, which limits generalizability.

## Conclusion

This study demonstrates that serum lactate, especially when interpreted in combination with carboxyhemoglobin levels, is a strong and mechanistically grounded predictor of hyperbaric oxygen therapy requirement in acute carbon monoxide poisoning. By quantifying the individual and combined diagnostic performance of accessible laboratory parameters, we propose a practical and evidence-based framework to support early risk stratification in the emergency setting. The identification of clinically meaningful lactate thresholds further enhances the applicability of our findings. Future prospective studies are essential to validate this model and explore its integration into standardized triage protocols and clinical decision-support systems.

## Data Availability

The datasets used and analyzed during the current study are available from the corresponding author on reasonable request.

## Abbreviations

CO: Carbon monoxide
COHb: Carboxyhemoglobin
HBOT: Hyperbaric oxygen therapy
WBC: White blood cell (count)
NLR: Neutrophil—to—lymphocyte ratio
PLR: Platelet—to—lymphocyte ratio
MPV: Mean platelet volume
RDW: CV—Red cell distribution width—coefficient of variation
NEU: Neutrophil (count)
LYM: Lymphocyte (count)
PLT: Platelet (count)
PSS: Poison Severity Score
OR: Odds ratio
CI: Confidence interval
ROC: Receiver operating characteristic
AUC: Area under the curve
LRT: Likelihood—ratio test
AIC: Akaike information criterion
BIC: Bayesian information criterion
CBC: Complete blood count
